# Paid Leave and Access to Telework as Work Attendance Determinants during Acute Respiratory Illness, United States, 2017–2018

**DOI:** 10.3201/eid2601.190743

**Published:** 2020-01

**Authors:** Faruque Ahmed, Sara Kim, Mary Patricia Nowalk, Jennifer P. King, Jeffrey J. VanWormer, Manjusha Gaglani, Richard K. Zimmerman, Todd Bear, Michael L. Jackson, Lisa A. Jackson, Emily Martin, Caroline Cheng, Brendan Flannery, Jessie R. Chung, Amra Uzicanin

**Affiliations:** Centers for Disease Control and Prevention, Atlanta, Georgia, USA (F. Ahmed, S. Kim, B. Flannery, J.R. Chung, A. Uzicanin);; University of Pittsburgh, Pittsburgh, Pennsylvania, USA (M.P. Nowalk, R.K. Zimmerman, T. Bear);; Marshfield Clinic Research Institute, Marshfield, Wisconsin, USA (J.P. King, J.J. VanWormer);; Texas A&M University, Temple, Texas, USA (M. Gaglani);; Kaiser Permanente Washington Health Research Institute, Seattle, Washington, USA (M.L. Jackson, L.A. Jackson);; University of Michigan, Ann Arbor, Michigan, USA (E. Martin, C. Cheng)

**Keywords:** acute respiratory illness, influenza, pandemics, sick leave, sick days, illness days, productivity, telecommute, organizational policy, viruses, United States, telework, paid leave, work attendance

## Abstract

We assessed determinants of work attendance during the first 3 days after onset of acute respiratory illness (ARI) among workers 19–64 years of age who had medically attended ARI or influenza during the 2017–2018 influenza season. The total number of days worked included days worked at the usual workplace and days teleworked. Access to paid leave was associated with fewer days worked overall and at the usual workplace during illness. Participants who indicated that employees were discouraged from coming to work with influenza-like symptoms were less likely to attend their usual workplace. Compared with workers without a telework option, those with telework access worked more days during illness overall, but there was no difference in days worked at the usual workplace. Both paid leave benefits and business practices that actively encourage employees to stay home while sick are necessary to reduce the transmission of ARI and influenza in workplaces.

## Abstract 2

Practices that actively encourage employees to stay home when ill are crucial to reduce transmission in workplaces.

The annual economic burden of influenza in the United States, depending on the severity of the influenza season, ranges from $15 billion to $64 billion, of which lost productivity accounts for a substantial proportion (*1*). The annual economic burden of noninfluenza viral respiratory tract infections is estimated to be $40 billion (*2*). As a result of absenteeism and diminished work capacity, employees with medically attended influenza can expect to lose 69% of their usual workplace productivity and employees with noninfluenza acute respiratory illness (ARI) can expect to lose 58% of their usual workplace productivity during the week after symptom onset (*3*). With about two thirds of the US adult population participating in the labor force (*4*), workplace contacts can play a major role in the transmission of influenza (*5*). Influenza vaccination can reduce illness and work absenteeism associated with influenza (*6*), but fewer than one third of US adults 18–64 years of age were vaccinated in the 2017–2018 influenza season (*7*).

Respiratory etiquette, regular hand hygiene, and staying home for >24 hours after fever subsides can help slow the spread of seasonal and pandemic influenza (*8*). For employed adults, staying home when ill usually entails taking sick days or working from home. During a respiratory illness, some employees may have a telework option, whereby they are permitted to perform their usual work functions while staying at home (without having to use paid time off or sick leave benefits). Telework may be a good mitigation strategy during an influenza pandemic if ill persons work remotely and avoid exposing co-workers during the contagious period (*9*). About 24% of employed persons in the United States telework regularly, varying from 8% in production occupations to 34% in managerial and professional occupations (*10*). Teleworking options also tend to track closely with education; 13% of workers with less than a high school diploma report being able to telework, compared with 37% of those with a bachelor’s degree or higher (*10*).

Approximately 74% of US civilian workers receive paid sick leave and 75% receive paid vacation leave benefits (*11*). However, the effect of access to telework and paid leave benefits on staying away from the workplace during influenza illness is largely unknown (*5*). This study assessed the association between access to telework and paid leave benefits and short-term work attendance in employed adults during a medically attended ARI or influenza episode.

## Methods

### Study Population

Study enrollees were patients seeking care for an ARI with cough within 7 days of illness onset during November 1, 2017–April 19, 2018 (the 2017–18 influenza season), at outpatient facilities affiliated with sites participating in the US Influenza Vaccine Effectiveness Network. The sites are in Ann Arbor and Detroit, Michigan; Pittsburgh, Pennsylvania; Temple, Texas; Seattle, Washington; and Marshfield, Wisconsin, USA. The study methods have been published previously (*12*,*13*). The institutional review boards at the sites approved the study. Study participants provided informed consent.

### Data Collection

Data collected at the enrollment visit included sex, race/ethnicity, education, general health before illness, number of children <12 years of age living in household, date of illness onset, and symptoms (fever/feverishness, sore throat) (Appendix Table 1). Data extracted from electronic medical records included age, medical conditions associated with increased risk of influenza complications (based on medical encounters associated with International Classification of Diseases codes in the year before enrollment) (*12*), and influenza vaccination. Nasal and oropharyngeal swab specimens were collected at the enrollment visit; all persons were tested for influenza viruses using real-time reverse transcription PCR (rRT-PCR).

Adults 19–64 years of age were asked to complete a survey 7–14 days after enrollment. The follow-up survey for the 2017–18 influenza season included questions about the following: hours expected to work in a typical week, hours usually worked from home (telework, telecommute, or remote work), receipt of any paid leave that could be used for an illness (e.g., sick leave, personal time off, vacation leave), whether they worked the day before illness, and work attendance during the first 3 days of illness (including number of days worked at the usual workplace and number of days worked from home) (Appendix Table 1). Participants were also asked about recovery from illness, return to normal activities (e.g., work, exercise, housework/chores), type of employee, type of position, and number of employees in the company/organization. Workers were asked to rate their level of agreement with 3 statements about their place of work using a Likert scale (Appendix Table 1). Responses were dichotomized as “agree” or “not agree”; “strongly agree” and “agree” responses were categorized as “agree.”

### Definitions

Study participants who reported that they regularly worked from home >1 hour in a typical week were classified as having access to telework (habitual teleworkers). Because persons who worked from home for only a few hours a week may not telework >1 full day a week, we performed an analysis based on the hours teleworked (none, <8 hours, or >8 hours) to assess the robustness of the findings (*14*). Persons who reported that they received any paid leave that could be used for an illness (e.g., paid sick leave, vacation leave, or personal time off) were classified as having paid leave benefit (*15*). Part-time workers were those working >20 but <35 hours; full-time was defined as >35 hours/week (*10*). We computed the total number of days worked in the first 3 days of illness by summing the number of days worked at the usual workplace and the number of days worked from home. We defined laboratory-confirmed influenza as a positive rRT-PCR test for influenza A or B from a nasal or oropharyngeal swab specimen.

### Inclusion and Exclusion Criteria

If an adult 19–64 years of age enrolled in the study >2 times because of multiple episodes of ARI during the influenza season, we included the first enrollment (Figure). Participants who completed the follow-up survey >14 days after enrollment were excluded to minimize recall bias (*16*). Participants were also excluded if they were unemployed, self-employed, owned their own business, worked solely from home, or were employed <20 hours/week. Only responses that added up to 3 days for the question on work attendance during the first 3 days of illness were considered valid and used for the final analysis. Examples of valid and invalid responses are provided in Appendix Table 2.

**Figure:**
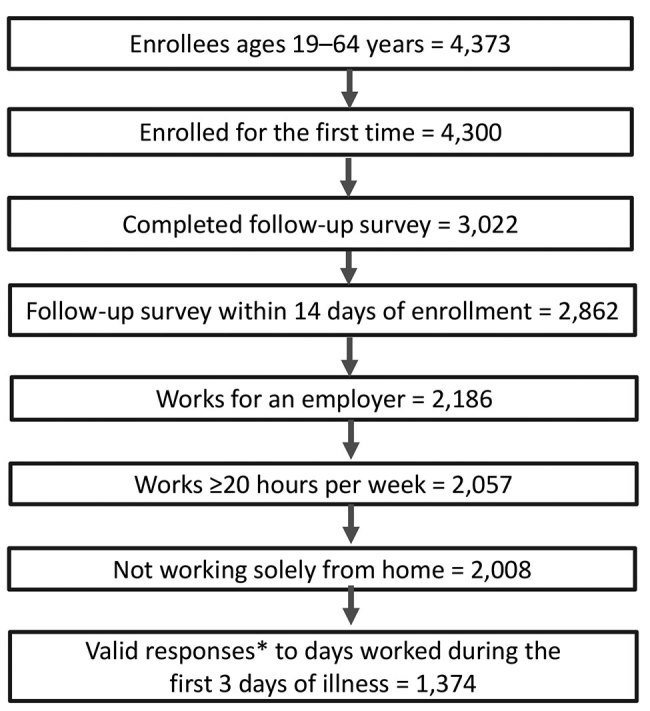
Total enrolled and number of persons included in analyses of work attendance during the first 3 days of acute respiratory illness or influenza, United States, 2017–18 influenza season. *Valid responses are those that added up to 3 days for the question on work attendance during the first 3 days of illness (see Appendix Table 2).

### Statistical Analysis

We computed the median days to return to normal activities and the median days to recovery from illness using the Kaplan-Meier method to take into account that a substantial proportion of participants had not returned to normal activities (13%) or recovered from their illness (32%) at the time of follow-up. We used a χ^2^ test to assess differences between categories, Student *t*-test for means, and Wilcoxon rank-sum tests to assess differences between medians. We ran zero-inflated Poisson regression, which accounts for excess zeroes, using PROC GENMOD in SAS version 9.4 (SAS Institute, https://www.sas.com) to compute ratios of days worked adjusted for potential confounding variables (*17*). The dependent variable in the regression models was the number of days worked during the first 3 days of illness (i.e., 0, 1, 2, or 3 days). In addition to running models using the total days worked during the first 3 days of illness, an indicator of productivity, as the dependent variable, we ran models using days worked at the usual workplace during the first 3 days of illness, a measure of the potential for virus transmission to co-workers, as the dependent variable. The exposure variables were access to telework (0 = no, 1 = yes) and access to paid leave (0 = no, 1 = yes). We used a forward selection process. Because age, sex, and socioeconomic status have been identified as confounders (*18*), we retained age, sex, and education (proxy for socioeconomic status) in the models. Statistical significance was set at α = 0.05 (2-tailed).

## Results

A total of 4,300 adults 19–64 years of age were enrolled across the study sites. Overall, 2,862 (67%) adults completed the follow-up survey within 14 days of enrollment (Figure), and 2,008 workers met the employment criteria. Of these, 1,374 (68%) had valid responses for the question on work attendance during the first 3 days of illness and were included in the analysis. The proportion of adults with valid responses was 85% (757/894) for the Washington and Wisconsin sites and 55% (617/1,114) for the other 3 sites. The proportion with valid responses was higher for non-Hispanic whites and other races compared with non-Hispanic blacks and Hispanics (71% vs. 54%; p<0.001) and for hourly workers compared with non–hourly workers (71% vs. 66%; p<0.05). Valid responses were also higher for persons who received influenza vaccination (72% vs. 65%; p<0.01) and those with paid leave benefits (70% vs. 64%; p<0.05). The proportion with valid responses did not differ by age, sex, education, access to telework, and other variables (data not shown).

Among the 1,374 adults with valid responses, the median age was 42 years, 64% were female, and 82% were non-Hispanic white. The median hours expected to work in a typical week was 40 (5th, 95th percentile: 25, 52). Thirty-six percent of participants had laboratory-confirmed influenza; patients with influenza were more likely to report fever (84%) than patients who tested negative for influenza (53%) (p<0.001), whereas the proportions reporting sore throat were similar (77% vs. 81%). In accordance with the criteria for enrollment in the study, all participants’ symptoms included cough. 

The median time from illness onset to enrollment was 3 days (5th, 95th percentile: 1, 7), and the median interval from enrollment to follow-up was 7 days (5th, 95th percentile: 6, 12). Based on the Kaplan-Meier method, the median interval from illness onset to return to normal activities was 7 days, and the median interval from illness onset to recovery from illness was 11 days. During the first 3 days of illness, 539 (39%) reported that they did not work at all. The mean number of total days worked during the first 3 days of illness was 1.14 days (range 0–3 days), the mean number of days absent from work because of illness was 1.06 days (range 0–3 days), and the mean number of days not worked because of having a scheduled day off or any other reason was 0.80 days (range 0–3 days). 

Data on access to telework were available for 1,362 adults and data on paid leave benefits for 1,356 adults; 198 (15%) reported having access to telework, and 1,074 (79%) received paid leave benefits. Among persons with access to telework, the median hours usually teleworked was 8 hours/week (5th, 95th percentile: 2, 30 hours/week). Adults who reported access to telework and paid leave were significantly different from those who reported no access by having higher education levels (p<0.001), working full time (p<0.01), and being salaried employees (p<0.001) (Table 1). Furthermore, adults with access to telework and paid leave were more likely to be encouraged by their employer to go home if they had influenza-like symptoms at work (p<0.01); these employees also had greater control over taking days off from work for illnesses (p<0.001) (Table 1).

**Table 1:** Characteristics associated with having access to telework and paid leave benefits among working adults with medically attended acute respiratory illness or influenza, United States, 2017–18 influenza season*

Characteristic	Access to telework			Paid leave benefits	
	Yes, n = 198	No, n = 1,164		Yes, n = 1,074	No, n = 282
Age, y, median (5th, 95th percentile)	43 (25, 61)	41 (22, 61)		**43§ (24, 61)**	**36 (20, 60)**
Sex					
F	119 (60)	756 (65)		695 (65)	175 (62)
M	79 (40)	408 (35)		379 (35)	107 (38)
Race/ethnicity					
White, non-Hispanic	**157 (79)†**	**956 (82)**		878 (82)	229 (81)
Black, non-Hispanic	**9 (5)**	**50 (4)**		46 (4)	11 (4)
Other race, non-Hispanic	**25 (13)**	**83 (7)**		89 (8)	23 (8)
Hispanic, any race	**7 (4)**	**72 (6)**		58 (5)	19 (7)
Education					
Some college or less	**45 (23)§**	**652 (56)**		**502 (47)§**	**188 (67)**
Bachelor’s or advanced degree	**153 (77)**	**509 (44)**		**571 (53)**	**92 (33)**
Current smoker					
Yes	**11 (6) ‡**	**148 (13)**		**102 (10)§**	**59 (21)**
No	**186 (94)**	**1,009 (87)**		**965 (90)**	**222 (79)**
General health before illness					
Excellent or very good	144 (73)	794 (68)		743 (69)	188 (67)
Good	44 (22)	308 (27)		282 (26)	71 (25)
Fair or poor	10 (5)	60 (5)		48 (4)	22 (8)
Fever/feverishness during illness					
Yes	128 (65)	742 (64)		694 (65)	172 (61)
No	70 (35)	422 (36)		380 (35)	110 (39)
Medical conditions associated with higher risk of influenza complications¶					
Yes	93 (47)	586 (50)		545 (51)	134 (48)
No	105 (53)	578(50)		529 (49)	148 (52)
Received influenza vaccine since July 1, 2017					
Yes	101 (51)	597 (51)		**604 (56)§**	**90 (32)**
No	97 (49)	567 (49)		**470 (44)**	**192 (68)**
Children <12 y of age in household					
0	136 (69)	772 (66)		713 (66)	192 (68)
1	33 (17)	195 (17)		180 (17)	47 (17)
>2	29 (15)	196 (17)		180 (17)	43 (15)
Worked the day before illness#					
Yes	124 (65)	741 (65)		689 (65)	177 (64)
No	68 (35)	405 (35)		366 (35)	100 (36)
Paid leave benefits					
Yes	**164 (85)†**	**900 (78)**		NA	NA
No	**30 (15)**	**250 (22)**		NA	NA
Access to telework					
Yes	NA	NA		**164 (15)†**	**30 (11)**
No	NA	NA		**900 (85)**	**250 (89)**
Employees are discouraged from coming to work when they have influenza-like symptoms**					
Agree	152 (78)	833 (72)		**797 (75)‡**	**186 (66)**
Not agree	44 (22)	327 (28)		**271 (25)**	**95 (34)**
Employees are encouraged to go home if they have influenza-like symptoms at work**					
Agree	**165 (84)‡**	**865 (75)**		**838 (79)§**	**188 (67)**
Not agree	**31 (16)**	**293 (25)**		**229 (21)**	**92 (33)**
I have a lot of control over when I can take days off from work for illnesses**					
Agree	**171 (87)§**	**753 (65)**		**766 (72)§**	**152 (54)**
Not agree	**25 (13)**	**403 (35)**		**298 (28)**	**129 (46)**
Full-time worker					
Yes	**182 (92)‡**	**983 (84)**		**987 (92)§**	**173 (61)**
No	**16 (8)**	**181 (16)**		**87 (8)**	**109 (39)**
Employee type					
Hourly	**34 (18)§**	**761 (66)**		**556 (52)§**	**236 (84)**
Salaried or other	**159 (82)**	**397 (34)**		**509 (48)**	**44 (16)**
Executive, administrator, or senior manager position (if salaried)					
Yes	44 (30)	109 (29)		141 (29)	12 (44)
No	105 (70)	263 (71)		352 (71)	15 (56)
No. employees in organization					
<99	62 (34)	315 (30)		**232 (24)§**	**143 (58)**
100–499	29 (16)	181 (17)		**172 (18)**	**37 (15)**
>500	89 (49)	552 (53)		**573 (59)**	**67 (27)**
Study site					
Michigan	**38 (19)§**	**83 (7)**		**97 (9)§**	**26 (9)**
Pennsylvania	**44 (22)**	**257 (22)**		**219 (20)**	**81 (29)**
Texas	**20 (10)**	**169 (15)**		**150 (14)**	**32 (11)**
Washington	**78 (39)**	**295 (25)**		**328 (31)**	**46 (16)**
Wisconsin	**18 (9)**	**360 (31)**		**280 (26)**	**97 (34)**

*Values are no. (%) unless otherwise indicated. Boldface indicates statistical significance. Numbers may not sum to n because of missing data. NA, not applicable. †p<0.05. ‡p<0.01. §p*<*0.001. ¶Based on International Classification of Diseases codes in electronic medical records in the year before enrollment. #Among those who worked the day before illness, the proportion who teleworked was 14% (17/124) for those with access to telework and 1% (5/741) for those without telework access (p<0.001). The proportion who teleworked was 3% (18/689) for those with access to paid leave and 2% (4/177) for those without access to paid leave. Among those who did not work the day before illness, the proportion who did not work because it was a day off was 81% (55/68) for those with telework access, and 83% (336/405) for those without telework access. **”Strongly agree” and “agree” responses were coded as agree. “Strongly disagree,” “disagree,” and “neither agree nor disagree” responses were coded as not agree.

The proportion of adults who worked the day before illness was similar for those with access to telework compared with those without access, as well as for those with paid leave benefits compared with those without (Table 1). Among adults who worked the day before illness, telework was more common among those with access to telework (habitual teleworkers) than for those who were not habitual teleworkers (14% vs. 1%, p<0.001).

During the first 3 days of illness, the proportion who did not work at all was 28% (55/198) for those with access to telework compared with 41% (477/1,164) for those without telework access (p<0.001). The mean of the total days worked was greater for adults with access to telework than for adults without access to telework (mean 1.46 vs. 1.09 days; p<0.001) (Table 2). This difference was attributable to more days teleworking while ill, as there was no difference in the mean number of days worked at the usual workplace while ill (Table 2). Adults without access to telework took more time off because of illness (mean 1.10 vs. 0.80 days; p<0.001). In contrast, adults with access to paid leave showed no differences in the mean total days worked during acute illness or mean days worked at the usual workplace, compared with those among persons without access to paid leave (Table 2).

**Table 2:** Work attendance during the first 3 days of illness among adults with medically attended acute respiratory illness or influenza, United States, 2017–18 influenza season*

Work attendance	Mean no. days worked				
	Access to telework			Paid leave benefits	
	Yes, n = 198	No†, n = 1,164		Yes, n = 1,074	No, n = 282
Worked	**1.46‡**	**1.09**		1.15	1.09
Usual workplace	1.05	1.07		1.07	1.05
Teleworked	**0.41‡**	**0.02**		0.08	0.04
Did not work	**1.54‡**	**1.91**		1.85	1.91
Felt ill	**0.80‡**	**1.10**		1.03	1.17
Day off	0.64	0.72		0.72	0.66
Other reasons	0.11	0.10		0.10	0.07

*Days worked or not worked ranged from 0 to 3 days. Boldface indicates statistical significance. †Among 1,164 persons with no telework access (i.e., did not habitually telework), 15 persons reported that they worked from home for ≥1 d during the first 3 d of illness. ‡p<0.001.

The results of zero-inflated Poisson regression analyses showed that participants who had access to paid leave were significantly less likely to work during the first 3 days of illness (adjusted ratio of days worked 0.81, 95% CI 0.68–0.96) or to work at their usual workplace (adjusted ratio of days worked 0.81, 95% CI 0.67–0.96) (Table 3). Persons who worked in an organization in which employees were discouraged from coming to work if they had influenza-like symptoms were also significantly less likely to work during the first 3 days of illness (adjusted ratio 0.86, 95% CI 0.76–0.97) or to work at their usual workplace (adjusted ratio 0.85, 95% CI 0.74–0.96) (Table 3). In contrast, persons with access to telework were significantly more likely to work during the first 3 days of illness (adjusted ratio 1.25, 95% CI 1.07–1.46) (Table 3). However, access to telework was not associated with the number of days worked at the usual workplace (adjusted ratio 0.98, 95% CI 0.82–1.17) (Table 3).

**Table 3:** Adjusted analysis to assess the association with days worked during the first 3 days of illness among adults with medically attended acute respiratory illness or influenza, United States, 2017–18 influenza season*

Characteristic	Total days worked, n = 1,306	Days worked at the usual workplace, n = 1,306
Access to telework		
No	Referent	Referent
Yes	**1.25 (1.07–1.46)†**	0.98 (0.82**–**1.17)
Access to paid leave		
No	Referent	Referent
Yes	**0.81 (0.68–0.96)‡**	**0.81 (0.67–0.96)‡**
Discouraged from coming to work with influenza-like symptoms		
Not agree	Referent	Referent
Agree	**0.86 (0.76–0.97)‡**	**0.85 (0.74–0.96)‡**

*Values are adjusted ratios of days worked (95% CI). Boldface indicates statistical significance. Total days worked represents the sum of days worked at the usual workplace and days teleworked during the first 3 days of illness. The dependent variable in the zero-inflated Poisson regressions was days worked during the first 3 days of illness (i.e., 0, 1, 2, or 3 d). The final models contained the following independent variables: access to telework; access to paid leave; employees are discouraged from coming to work when they have flulike symptoms; age; sex; education; fever; worked the day before illness; having a lot of control over taking days off for illnesses; full-time worker; and employee type. The variable “employees are encouraged to go home if they have influenza-like symptoms at work” was excluded from the models because it was highly correlated with the variable “employees are discouraged from coming to work when they have influenza-like symptoms” (Spearman correlation coefficient 0.76; p<0.001); the latter variable has more relevance for reducing virus transmission in the workplace (not coming to work at all vs. coming to work with influenza-like symptoms and then told to go home). Sixty-eight records were excluded because of missing values. †p<0.01. ‡p<0.05.

The findings were similar among workers with laboratory-confirmed influenza (Table 4; Appendix Tables 3–5). Results were similar for sites with higher proportions of valid responses (Washington and Wisconsin) and lower proportions (Michigan, Pennsylvania, and Texas sites) (Appendix Table 6). The analysis by hours teleworked showed similar findings (Appendix Table 7).

**Table 4:** Adjusted analysis to assess the association with days worked during the first 3 days of illness, United States, 2017–18 influenza season, by laboratory-confirmed influenza*

Characteristic	Total days worked				Days worked at the usual workplace	
Influenza positive, n = 464	Influenza negative, n = 839		Influenza positive, n = 464	Influenza negative, n = 839
Access to telework						
No	Referent	Referent			Referent	Referent
Yes	**1.46 (1.09–1.96)†**	1.19 (0.99**–**1.43)			1.15 (0.83**–**1.60)	0.92 (0.75**–**1.14)
Access to paid leave						
No	Referent	Referent			Referent	Referent
Yes	0.81 (0.57**–**1.14)	0.82 (0.68**–**1.00)			0.79 (0.55**–**1.12)	0.83 (0.68**–**1.02)
Discouraged from coming to work with influenza-like symptoms						
Not agree	**Referent**	Referent			**Referent**	Referent
Agree	**0.71 (0.55–0.91)‡**	0.92 (0.80**–**1.06)			**0.72 (0.55–0.94)†**	0.90 (0.78**–**1.05)

*Data are presented as adjusted ratios of days worked (95% confidence interval), unless otherwise indicated. Boldface indicates statistical significance. The dependent variable in the zero-inflated Poisson regressions was the number of days worked during the first 3 days of illness. The final models contained the following independent variables: access to telework; access to paid leave; employees are discouraged from coming to work when they have influenza-like symptoms; age; sex; education; fever; worked the day before illness; having a lot of control over taking days off for illnesses; full-time worker; and employee type. Sixty-eight records were excluded because of missing values, and an additional 3 records were excluded because laboratory confirmation of influenza by real-time reverse transcription PCR was not available. †p<0.05. ‡p<0.01.

## Discussion

Among working adults who sought medical care for an ARI from 5 sites across the country, we found that 79% had access to paid leave and 15% were able to telework. Our study results show that both paid leave benefits and business practices that actively encourage employees to stay home when ill may be necessary to keep sick employees away from the workplace. Access to telework, where feasible, helps retain some work productivity.

Because infectiousness of adults with influenza is greatest during the first 3 days of illness (*19**,**20*), preventing workplace attendance of ill persons during the first several days of illness might be most necessary for reducing workplace-based transmission. In previous research, a greater proportion of workers reported going to work always or most of the time when they have a cold or influenza, compared with those experiencing more serious illnesses, injuries, or major physical problems (*21*). Reasons for working while experiencing influenza-like illness (ILI) include still being able to perform job duties, not feeling bad enough to miss work, not thinking their illness is contagious or could make other persons sick, and professional obligation to co-workers (*22*). We have documented that workplace cultures that encourage employees to refrain from coming to work when ill may play a crucial role in keeping workers away from the workplace when sick. In this study, persons with access to paid leave worked fewer days overall and at the usual workplace while ill. Two previous studies reported that access to paid sick days was associated with staying home for medically confirmed ILI or influenza (*23**,**24*), and 1 study found no association between having paid sick leave benefits and staying home from work because of ILI (*25*). These 3 studies did not assess telework.

We found that workers with access to telework used this benefit to work more total days while ill than those without it. Access to telework may enable persons to work from home on a day that they might otherwise have to take a sick day to comply with the “stay home when sick” recommendation. Availability of telework options is therefore possibly beneficial from the employer’s perspective in terms of reduced sick leave usage and preserved productivity. However, we observed little difference between workers who have access to telework and those who do not regarding the number of days worked at the usual workplace while sick. This finding suggests that just having telework policies in place may not be sufficient to keep workers with access to telework from going to their workplace while sick. More effort is needed to encourage sick workers with telework access to work from home instead of at their usual workplace. In contrast to our findings with regard to telework, a previous study of workers in 3 large US companies (a national retail chain, a transportation company, and a durable goods manufacturing company) during the 2007–08 influenza season reported that workers who could telework had a 30% lower rate of attending work at their usual worksite when they had severe ILI symptoms (*26*). However, the authors acknowledged that their study was based on a convenience sample of only 3 employers, which limited the generalizability of their findings.

The 2017–18 influenza season, during which influenza A(H3N2) viruses predominated, was a high-severity season with widespread influenza activity across the country for an extended period (*27*). The influenza A(H3N2) strain typically causes more severe symptoms than the influenza A(H1N1) strain (*28*). The health-related workplace absenteeism rate in the 2017–18 influenza season was higher than the average rate of the previous 5 seasons (*29*). It is unknown whether the findings of this study would be similar in a less-severe influenza season. However, our results were similar for influenza-negative ARI cases, which are usually less severe than influenza cases. Thus, it seems likely that the findings would be similar in less-severe influenza seasons.

Our study has some limitations. First, almost one third of eligible adults were excluded from the analysis because of invalid responses regarding work attendance during the first 3 days of illness. However, similar results were seen for the Washington and Wisconsin sites, which had higher rates of valid responses than the other 3 sites. Second, we assessed work attendance during the first 3 days of illness. Further research on work attendance during the subsequent days of illness may be helpful. Third, our study was conducted among workers with medically attended ARI. The findings may not be generalizable to workers with non–medically attended ARI, which tends to be less severe (*30*). Fourth, our study indicates that employees with access to telework worked more days overall than those without telework access. We did not, however, assess actual levels of workplace productivity or measures of output. Adults may have reduced work performance if they worked, whether on-site or remotely from home, while not feeling well (*13**,**31*). Fifth, our definition of paid leave included both paid sick days and paid vacation days. Because paid vacation leave may be less flexible than paid sick leave for taking time off on short notice for an unexpected illness (*15*), more research is needed to assess the effect of paid sick days on work attendance among persons with ARI or influenza. Finally, although we adjusted for potential confounding variables, an observational study such as ours cannot rule out the possibility that unmeasured variables (e.g., occupation) may have distorted the results. However, the proportion who worked the day before illness, which represents baseline measurement of the outcome, was similar between the intervention and control groups (e.g., telework access versus no telework access), indicating that the groups were initially comparable with a lower likelihood of the presence of confounding variables (*32*).

The desired public health result of employee access to paid leave and telework is an increased ability to comply with the public health recommendation to stay home when ill, which helps decrease risk of disease transmission in the workplace. Ideally, staying home when ill with a respiratory infectious disease should eventually become commonplace behavior or even a social norm. Having access to paid leave is likely a critical enabling factor that reduces financial barriers to staying away from work when ill (*33**–**35*). Organizational policies that are conducive to providing paid leave are therefore critically needed, but almost equally crucial are supportive business practices that actively encourage employees to stay home when sick. Therefore, both broader macro-level policy interventions and stimulation of business culture change at a micro level of individual work organizations, possibly even individual teams, may be necessary to help reduce the transmission of ARI and seasonal or pandemic influenza in workplaces.

Appendix Additional information about paid leave and access to telework as work attendance determinants during acute respiratory illness.
