# Supplementary material for: Paid Leave and Access to Telework as Work Attendance Determinants during Acute Respiratory Illness, United States, 2017–2018
Source: Emerg Infect Dis. 2020 Jan;26(1):26–33. doi: 10.3201/eid2601.190743 (PMC6924903; doi:10.3201/eid2601.190743)
Supplement: Appendix — Additional information about paid leave and access to telework as work attendance determinants during acute respiratory illness. [file 19-0743-Techapp-s1.pdf]

Article DOI: https://doi.org/10.3201/eid2601.190743

Paid Leave and Access to Telework as Work Attendance Determinants during Acute Respiratory Illness, United States, 2017–2018

Appendix

Appendix Table 1. Selected enrollment and follow-up questions, 2017–2018 influenza season.

Form	Question	Values
Enrollment	When did your illness begin?	Date
	For this next question, please think about your general health before you got this current illness. In general, would you say that your health is excellent, very good, good, fair, or poor?	Excellent<br>Very good<br>Good<br>Fair<br>Poor
	Which of the following symptoms have you experienced since the illness began? (Check all that apply)	Fever/feverishness<br>Sore throat
Follow-up*	Are you currently employed (work for pay or profit)?	I work for an employer<br>I am self-employed or own my own business<br>No
	How many hours are you expected to work in a typical 7-day week? (If it varies, estimate the average.)	Hours
	Of those expected hours, how many hours in a week do you usually work from home (telework, telecommute, or remote work)? (Enter “0” if none)	Hours
	Do you receive any paid leave that could be used for an illness, such as sick leave, personal time off, or vacation leave?	Yes<br>No
	During the first 3 days of your illness, please record the number of days you:<br>- Did not work at all because it was a day off<br>- Did not work at all because you felt ill<br>- Did not work at all because of any other reason, including vacation<br>- Went to work at your usual workplace<br>- Worked from home (telework, telecommute, or remote work)	Days
	Did you work the day <u>before</u> you became ill?<br>- Did not work at all because it was a day off<br>- Did not work at all because of any other reason (including vacation)<br>- Went to work at your usual workplace<br>- Worked from home (telework, telecommute, or remote work)	Yes<br>No
	Please select your level of agreement with the following statements about your place of work.<br>- Employees are discouraged from coming to work when they have flulike symptoms.<br>- Employees are encouraged to go home if they have flulike symptoms at work.<br>- I have a lot of control over when I can take days off from work for illnesses.	Strongly agree<br>Agree<br>Neither agree nor disagree<br>Disagree<br>Strongly disagree

*Participants who worked multiple jobs were instructed to think about the job they considered their primary job.

Appendix Table 2. Examples of valid and invalid responses to survey question on work attendance during the first 3 days of illness, 2017–2018 influenza season

Examples
Example 1: Valid response (responses add up to 3 d)
- Did not work at all because it was a day off*: **2 d**
- Did not work at all because you felt ill: **1 d**
- Did not work at all because of any other reason, including vacation: **0 d**
- Went to work at your usual workplace: **0 d**
- Worked from home (telework, telecommute, or remote work): **0 d**
Example 2: Invalid response (responses add up to 5 d)
- Did not work at all because it was a day off*: **0 d**
- Did not work at all because you felt ill: **2 d**
- Did not work at all because of any other reason, including vacation: **0 d**
- Went to work at your usual workplace: **3 d**
- Worked from home (telework, telecommute, or remote work): **0 d**
*For persons who work 5 d/week from Monday through Friday, Saturday and Sunday would constitute days off.

Appendix Table 3. Association between access to telework and work attendance during the first 3 days of illness, 2017–2018 influenza season, by laboratory-confirmed influenza

Work attendance	Influenza positive*		Influenza negative	
Mean days worked	Telework access (n = 75)	No telework access (n = 413)	Telework access (n = 122)	No telework access (n = 749)
Worked	**1.21†**	**0.79**	**1.61†**	**1.26**
Usual workplace	0.87	0.77	1.16	1.23
Teleworked	**0.33‡**	**0.01**	**0.44‡**	**0.02**
Did not work	**1.79†**	**2.21**	**1.39†**	**1.74**
Felt ill	**1.02†**	**1.40**	**0.67†**	**0.93**
Day off	0.60	0.69	0.66	0.73
Other reasons	0.17	0.12	0.07	0.09

*Days worked or not worked ranged from 0 to 3 d. Boldface indicates statistical significance. Laboratory confirmation of influenza by rRT-PCR was not available for 3 adults.
†p < 0.01.
‡p < 0.001.

Appendix Table 4. Association between access to paid leave and work attendance during the first 3 days of illness, 2017–2018 influenza season, by laboratory-confirmed influenza

Work attendance	Influenza positive*		Influenza negative	
Mean days worked	Paid leave access (n = 390)	No paid leave access (n = 95)	Paid leave access (n = 681)	No paid leave access (n = 187)
Worked	0.86	0.87	1.32	1.20
Usual workplace	0.78	0.86	1.24	1.14
Teleworked	**0.07†**	**0.01**	0.09	0.06
Did not work	2.14	2.13	1.68	1.80
Felt ill	1.31	1.43	0.88	1.04
Day off	0.72	0.58	0.71	0.71
Other reasons	0.12	0.12	0.09	0.05

*Days worked or not worked ranged from 0 to 3 days. Boldface indicates statistical significance. Laboratory confirmation of influenza by rRT-PCR was not available for 3 adults.
†p < 0.01.

Appendix Table 5. Association between agreement with statement that employees are discouraged from coming to work when they have flu-like symptoms and work attendance during the first 3 days of illness, 2017–2018 influenza season, by laboratory-confirmed influenza*

Work attendance	Influenza positive†		Influenza negative		Total‡	
Mean days worked	Agree (n = 373)	Not agree (n = 115)	Agree (n = 619)	Not agree (n = 257)	Agree (n = 995)	Not agree (n = 372)
Worked	**0.77¶**	**1.10**	**1.24§**	**1.44**	**1.07#**	**1.33**
Usual workplace	**0.72§**	**1.01**	**1.16§**	**1.37**	**0.99#**	**1.26**
Teleworked	0.05	0.09	0.09	0.07	0.08	0.07
Did not work	**2.23¶**	**1.90**	**1.76§**	**1.56**	**1.93#**	**1.67**
Felt ill	1.38	1.19	**0.96§**	**0.77**	**1.12¶**	**0.90**
Day off	0.71	0.63	0.72	0.71	0.72	0.69
Other reasons	0.14	0.08	0.08	0.09	0.10	0.09

*Days worked or not worked ranged from 0 to 3 d. Boldface indicates statistical significance. "Strongly agree" and "agree" responses were coded as Agree. "Strongly disagree," "disagree," and "neither agree nor disagree" responses were coded as Not Agree.
†Laboratory confirmation of influenza by rRT-PCR was not available for 3 adults.
‡For the day before illness, the proportion who worked was 64% for those who agreed compared with 68% for those who disagreed ($p = 0.12$).
§$p < 0.05$.
¶$p < 0.01$.
#$p < 0.001$.

Appendix Table 6. Adjusted analysis to assess the association with days worked during the first 3 days of illness among adults with medically attended illness, 2017–2018 influenza season, by study site*

	Total days worked		Days worked at the usual workplace	
Characteristic	WA and WI (n = 731)	MI, PA, and TX (n = 575)	WA and WI (n = 731)	MI, PA, and TX (n = 575)
Access to telework				
No	1.00 (Referent)	1.00 (Referent)	1.00 (Referent)	1.00 (Referent)
Yes	**1.33 (1.07–1.64)†**	1.18 (0.95–1.47)	0.94 (0.73–1.21)	1.00 (0.78–1.28)
Access to paid leave				
No	1.00 (Referent)	1.00 (Referent)	1.00 (Referent)	1.00 (Referent)
Yes	0.84 (0.66–1.07)	0.81 (0.63–1.04)	0.84 (0.66–1.08)	0.79 (0.61–1.03)
Discouraged from coming to work with flulike symptoms				
Not agree	1.00 (Referent)	1.00 (Referent)	1.00 (Referent)	1.00 (Referent)
Agree	**0.84 (0.71–0.99)†**	0.90 (0.75–1.08)	**0.81 (0.68–0.96)†**	0.90 (0.74–1.09)

*Data are presented as adjusted ratios of days worked (95% CI), unless otherwise indicated. Boldface indicates statistical significance. Total days worked represents the sum of days worked at the usual workplace and days teleworked. The dependent variable in the zero-inflated Poisson regressions was the number of days worked during the first 3 days of illness. The final models contained the following independent variables: access to telework; access to paid leave; employees are discouraged from coming to work when they have flulike symptoms; age; sex; education; fever; worked the day before illness; having a lot of control over taking days off for illnesses; full-time worker; and employee type. Sixty-eight records were excluded because of missing values.
†$p < 0.05$.

Appendix Table 7. Adjusted analysis to assess the association between access to telework and work attendance during the first 3 days of illness, 2017–2018 influenza season, by hours teleworked per week*

Characteristic	Total days worked (n = 1,306)	Days worked at the usual workplace (n = 1,306)
Access to telework		
No	1.00 (Referent)	1.00 (Referent)
Yes, <8 h	1.22 (0.98–1.52)	1.05 (0.82–1.35)
Yes, ≥8 h	**1.27 (1.06–1.54)†**	0.92 (0.73–1.16)
Access to paid leave		
No	1.00 (Referent)	1.00 (Referent)
Yes	**0.81 (0.69–0.97)†**	**0.80 (0.67–0.96)†**
Discouraged from coming to work with flulike symptoms		

Characteristic	Total days worked (n = 1,306)	Days worked at the usual workplace (n = 1,306)
Not agree	1.00 (Referent)	1.00 (Referent)
Agree	**0.86 (0.76–0.97)†**	**0.85 (0.74–0.96)†**

*Data are presented as adjusted ratios of days worked (95% CI), unless otherwise indicated. Boldface indicates statistical significance. Total days worked represents the sum of days worked at the usual workplace and days teleworked. The dependent variable in the zero-inflated Poisson regressions was days worked during the first 3 days of illness (i.e., 0, 1, 2, or 3 d). The final models contained the following independent variables: access to telework; access to paid leave; employees are discouraged from coming to work when they have flu-like symptoms; age; sex; education; fever; worked the day before illness; having a lot of control over taking days off for illnesses; full-time worker; and employee type. Sixty-eight records were excluded because of missing values.

†$p < 0.05$.
